# A Case of Delayed Myelopathy Caused by Atlantoaxial Subluxation without Fracture

**DOI:** 10.1155/2013/421087

**Published:** 2013-04-02

**Authors:** Ryo Takamatsu, Hiroshi Takahashi, Yuichiro Yokoyama, Fumiaki Terajima, Yasuhiro Inoue, Katsunori Fukutake, Akihito Wada

**Affiliations:** Department of Orthopaedic Surgery, Toho University School of Medicine, 6-11-1 Omori-nishi, Ota-ku, Tokyo 143-8541, Japan

## Abstract

We report a case of delayed myelopathy caused by atlantoaxial subluxation without fracture. The patient was a 38-year-old male who became aware of weakness in extremities. The patient had a history of hitting his head severely while diving into a swimming pool at the age of 14 years old. At that time, cervical spine plain X-ray images showed no fracture, and the cervical pain disappeared after use of a collar for several weeks. At his first visit to our department, X-ray images showed an unstable atlantoaxial joint. After surgery, weakness of the extremities gradually improved. At 6 months after surgery, bone union was completed and the symptoms disappeared. This case shows that atlantoaxial ligament injuries are difficult to diagnose and may easily be missed. A high level of suspicion is important in such cases, since neurological compromise or deterioration may occur many years after the injury.

## 1. Introduction

Upper cervical spine injuries are rare in the pediatric population, comprising between 0.6 and 9.5% of all cervical injuries [1], and neurologic disability related to pediatric cervical spine injury is uncommon. Patel et al. found that only one-third of children with these injuries had a neurological deficit and half of these cases showed no initial evidence of a radiological anomaly [2]. Many patients with a spinal cord injury without radiographic abnormality show symptoms immediately after injury, but some symptoms may also develop after a latency period of several days [3]. Here, we report a rare case of myelopathy caused by atlantoaxial subluxation without fracture, in which symptoms developed after a 24-year latency period. We describe the case in the context of a discussion of the literature.

## 2. Case Report

The patient was a 38-year-old male who became aware of weakness in the right side of the body for several months without a specific trigger. He also developed motor skills disorder in his right hand that made it difficult for him to drive a car. Weakness in his left lower extremity also gradually developed and he then visited the Department of Neurology at our hospital. At this visit, intracranial process was denied, but hyperesthesia of the right upper and lower extremities was confirmed. Since no other abnormal findings were observed, he was referred to our department for suspected cervical spine pathology.

The patient had a history of hospital visits for several weeks for cervical pain after he hit the top of his head severely while diving into a swimming pool at the age of 14 years old. At that time, cervical spine plain X-ray images showed no fracture and he was diagnosed with whiplash injury. After the use of a collar for several weeks, the cervical pain disappeared.

**Figure 1:**
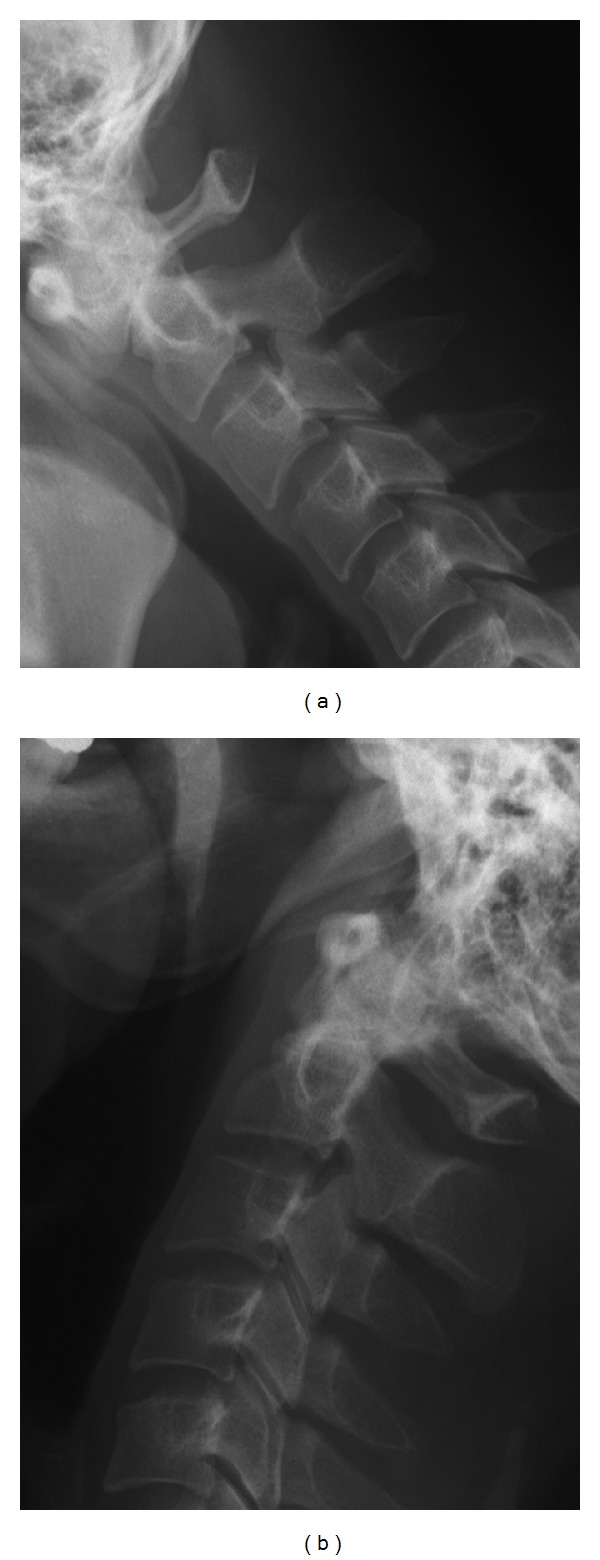
Plain X-ray images at the first visit under (a) flexion and (b) extension. Instability of the atlantoaxial joint was observed, with ADI increasing to 8 mm under flexion and reducing to ≤3 mm in extension.

**Figure 2:**
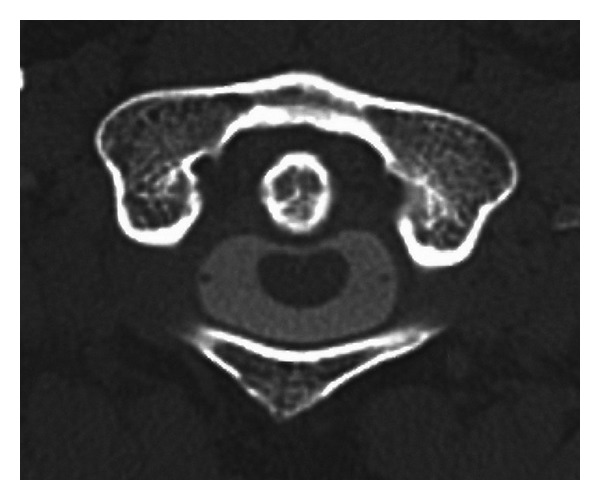
CT images after myelography. An increased ADI was apparent without evidence of fracture at the attachment site of the transverse ligament.

**Figure 3:**
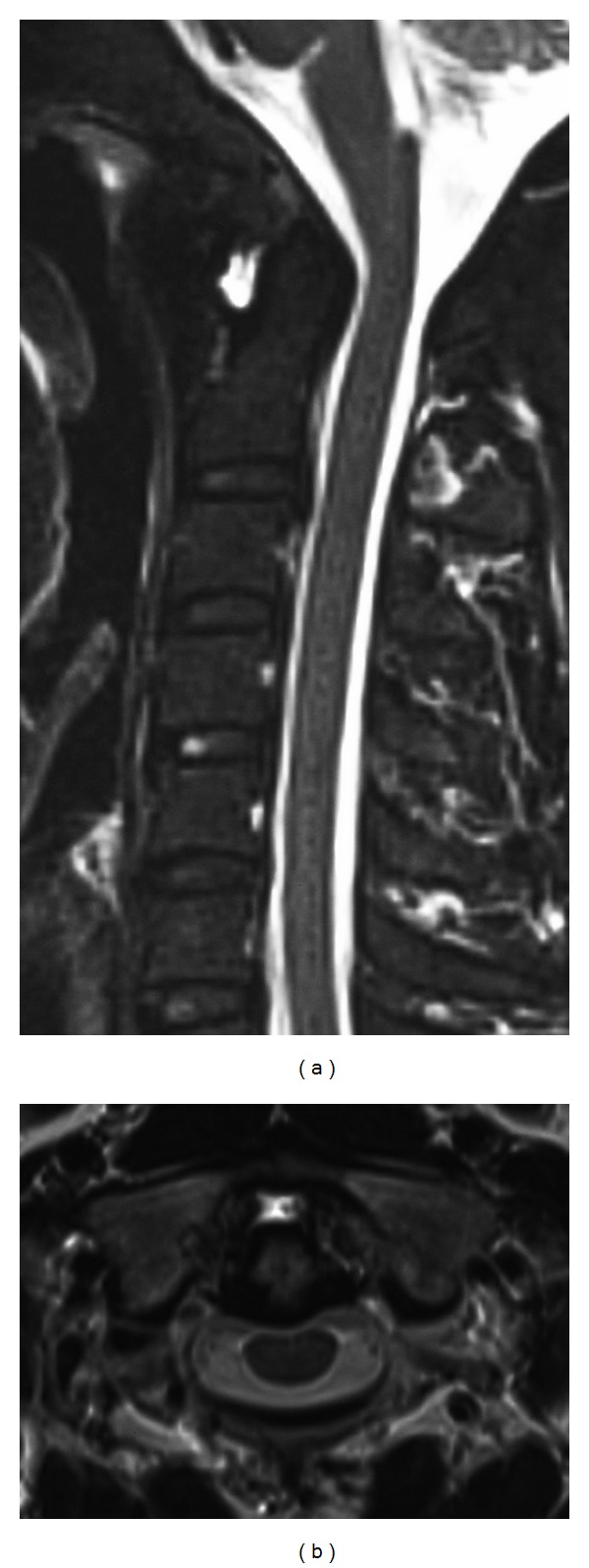
Cervical spine MRI. In (a) sagittal and (b) axial T2-weighted images, the high intensity area suggested fluid accumulation between the anterior arch of the atlas and odontoid process of the epistropheus.

At his first visit to our department, his right and left grip strengths were 47 kg and 42 kg, respectively, and no objective muscular depression was apparent in the extremities. There was also no increased deep tendon reflex or pathological reflex. Cervical spine plain X-ray images showed no fracture but indicated an unstable atlantoaxial joint. The atlantodental interval (ADI) was increased to 8 mm during flexion and reduced to ≤3 mm in extension (Figure 1). Computed tomography (CT) showed no bone chips at the attachment site of the transverse ligament of the atlas (Figure 2). Magnetic resonance imaging (MRI) showed a high intensity area, which suggested fluid accumulation caused by instability between the anterior arch of the atlas and odontoid process of the axis (Figure 3).

Based on this information, we diagnosed delayed myelopathy due to atlantoaxial subluxation caused by the injury of the transverse ligament of the atlas. Transarticular screw fixation surgery was performed under X-ray fluoroscopic guidance with the atlantoaxial joint in a hat-hook position. After the decortication of the posterior arch of the atlas and lamina of the vertebral arch axis, autologous iliac bone grafting was performed.

**Figure 4:**
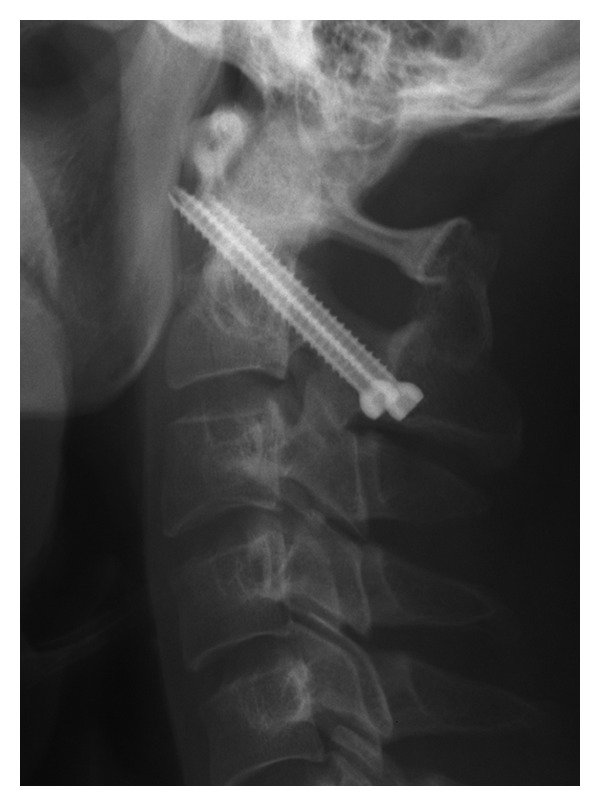
Postoperative cervical spine plain X-ray images. Atlantoaxial subluxation was improved with the adhesion of grafted bone.

The postoperative clinical course was uneventful. 4 days after surgery, walking exercises were initiated using a Philadelphia collar. Weakness of the extremities and motor skills disorder gradually improved. The patient was able to go back to work 3 months after surgery and showed no symptoms 6 months after surgery. Cervical spine plain X-ray images obtained 6 months after surgery showed union of grafted bone and no unstable cervical vertebra during flexion and extension (Figure 4).

## 3. Discussion

The incidence of blunt cervical spine injuries in children is 1% to 2% of all trauma victims requiring hospital admission [4]. The injury level of the cervical spine injury differs depending on age, with upper cervical injury being more common in younger children (0 to 8 years old), while lower cervical spine injury tends to occur more in older children (≥9 years old) [5, 6]. Upper cervical spine injuries in children are fundamentally different from those in adults because of anatomical differences in the developing spine. In children, the mass of the head is disproportionately large and the neck muscles are relatively underdeveloped. Furthermore, the vertebral bodies are wedge-shaped, the articulating facets are angled horizontally, the end plates are cartilaginous, and the interspinous ligaments are elastic and lax [7, 8]. These features predispose children to upper cervical spine injuries. Fewer fractures occur in children <8 years old compared to those ≥8 years old because of the greater mobility of the spine and the laxity of the ligaments in younger children. The vertebrae start to ossify and mature at 8 years old. Anteriorly, the vertebral body loses its wedge shape and becomes more rectangular, the orientation of the facets becomes less horizontal and more vertical, and the uncinate process begins to protrude [9–12].

Atlantoaxial subluxation accompanies fracture of the odontoid process of the axis in many cases. Most fractures of the odontoid in younger children occur at the dens-body synchondrosis, the weakest area of C2 [13]. Traumatic rupture of the transverse ligament as a cause of anatomical feature is extremely rare in children. Mechanisms of injury to the ligament include forced forward flexion and axial loading of the atlas, which opens the ring and causes secondary rupture of the transverse ligament, as in a Jefferson fracture with atlantoaxial subluxation. However, based on the history of injury and imaging findings in our case, we concluded that traumatic injury of the transverse ligament of the atlas occurred after the patient dived into a swimming pool when he was 14 years old.

Based on their experience in 4 patients aged 2–9 years old, Floman et al. [14] suggested that conservative treatment is not effective for atlantoaxial subluxation caused by injury of the transverse ligament of the atlas, and that surgical treatment should be performed. Dickman et al. [15] also suggested that early surgery is indicated in children aged ≥14 years old with a type 1 injury of the transverse ligament, whereas conservative treatment with a rigid cervical orthosis is preferred in patients with a type 2 injury with bone fragments in the ligament attachment; however, failure to achieve bone adhesion with such conservative treatment should be corrected with surgical treatment.

CT findings obtained at the first visit showed no evidence of fracture at the attachment site of the transverse ligament in our case, indicating a type 1 injury in the Dickman classification. Since the patient showed no neurological symptoms and complained only of cervical pain at the time of the initial trauma, the injury to the transverse ligament of the atlas may have been missed. There have been no previous reports of cases with such a long latency period, but we suggest that myelopathy in our case developed due to microdamage to the spinal cord caused by an unstable atlantoaxial joint at 24 years after the initial injury. This case shows that atlantoaxial ligament injuries are difficult to diagnose and may easily be missed. A high level of suspicion is important in such cases, since neurological compromise or deterioration may occur many years after the injury.

## References

[1] Kalfas I, Wilberger J, Goldberg A, Prostko ER (1988). Magnetic resonance imaging in acute spinal cord trauma. *Neurosurgery*.

[2] Patel JC, Tepas JJ, Mollitt DL, Pieper P (2001). Pediatric cervical spine injuries: defining the disease. *Journal of Pediatric Surgery*.

[3] Pang D, Wilberger JE (1982). Spinal cord injury without radiographic abnormalities in children. *Journal of Neurosurgery*.

[4] Dietrich AM, Ginn-Pease ME, Bartkowski HM, King DR (1991). Pediatric cervical spine fractures: predominately subtle presentation. *Journal of Pediatric Surgery*.

[5] Brown RL, Brunn MA, Garcia VF (2001). Cervical spine injuries in children: a review of 103 patients treated consecutively at a level 1 pediatric trauma center. *Journal of Pediatric Surgery*.

[6] Kokoska ER, Keller MS, Rallo MC, Weber TR (2001). Characteristics of pediatric cervical spine injuries. *Journal of Pediatric Surgery*.

[7] Apple JS, Kirks DR, Merten DF, Martinez S (1987). Cervical spine fractures and dislocations in children. *Pediatric Radiology*.

[8] Dickman CA, Zabramski JM, Hadley MN, Rekate HL, Sonntag VKH (1991). Pediatric spinal cord injury without radiographic abnormalities: report of 26 cases and review of the literature. *Journal of Spinal Disorders*.

[9] Fesmire FM, Luten RC (1989). The pediatric cervical spine: developmental anatomy and clinical aspects. *Journal of Emergency Medicine*.

[10] Hamilton MG, Myles ST (1992). Pediatric spinal injury: review of 61 deaths. *Journal of Neurosurgery*.

[11] Kewalramani LS, Kraus JF, Sterling HM (1980). Acute spinal-cord lesions in a pediatric population: epidemiological and clinical features. *Paraplegia*.

[12] Manary MJ, Jaffe DM (1996). Cervical spine injuries in children. *Pediatric Annals*.

[13] Buhs C, Cullen M, Klein M, Farmer D (2000). The pediatric trauma C-spine: is the ’odontoid’ view necessary?. *Journal of Pediatric Surgery*.

[14] Floman Y, Kaplan L, Elidan J, Umansky F (1991). Transverse ligament rupture and atlanto-axial subluxation in children. *Journal of Bone and Joint Surgery B*.

[15] Dickman CA, Greene KA, Sonntag VKH (1996). Injuries involving the transverse atlantal ligament: classification and treatment guidelines based upon experience with 39 injuries. *Neurosurgery*.

